# Dicom Color Medical Image Compression using 3D-SPIHT for Pacs Application

**Published:** 2008-06

**Authors:** T. Kesavamurthy, Subha Rani

**Affiliations:** *Department of ECE, PSG College of Technology, Peelamedu, Coimbatore, India*

**Keywords:** biorthogonal, dicom, JPEG 2000, PACS, 3D-SPIHT, wavelet

## Abstract

The proposed algorithm presents an application of 3D-SPIHT algorithm to color volumetric dicom medical images using 3D wavelet decomposition and a 3D spatial dependence tree. The wavelet decomposition is accomplished with biorthogonal 9/7 filters. 3D-SPIHT is the modern-day benchmark for three dimensional image compressions. The three-dimensional coding is based on the observation that the sequences of images are contiguous in the temporal axis and there is no motion between slices. Therefore, the 3D discrete wavelet transform can fully exploit the inter-slices correlations. The set partitioning techniques involve a progressive coding of the wavelet coefficients. The 3D-SPIHT is implemented and the Rate-distortion (Peak Signal-to-Noise Ratio (PSNR) vs. bit rate) performances are presented for volumetric medical datasets by using biorthogonal 9/7. The results are compared with the previous results of JPEG 2000 standards. Results show that 3D-SPIHT method exploits the color space relationships as well as maintaining the full embeddedness required by color image sequences compression and gives better performance in terms of the PSNR and compression ratio than the JPEG 2000. The results suggest an effective practical implementation for PACS applications.

## INTRODUCTION

Advanced medical imaging technologies, such as computed tomography (CT), magnetic resonance imaging (MRI) and traditional radiography are fundamental tools for providing more efficient and effective healthcare systems and services. The key to proliferation of these technologies is the digital representation of images. Digital medical images have potential benefits in terms of achieving and probability. In addition they offer versatility, enabling or expanding its applications in medical imaging (1). The volumetric color medical image refers to a regular 3D grid whose voxels contain RGB or grayscale information. The 3D medical images are derived from a discrete collection of samples generated by scientific simulations which is produced by computerized tomography (CT) medical imaging modality. The data sets used for the proposed algorithm are volumetric color medical images whose voxels contain the RGB information. A voxel is the smallest cubical structure. It has three axes for width, height and depth. A voxel has two parts (v, g) where v - location and g - pixel intensity. While using color images we need three intensity values for primary colors. The three-dimension image consists of a group of frames in which each frame is called rendition. The rendition consists of array of voxels with its depth indicating the gap between the renditions. Volume of Interest is the area most pertinent for the consideration. Medical volumetric data generated by CT typically contains many image slices that represent cross sections of a part of human anatomy.

**Figure 1:**
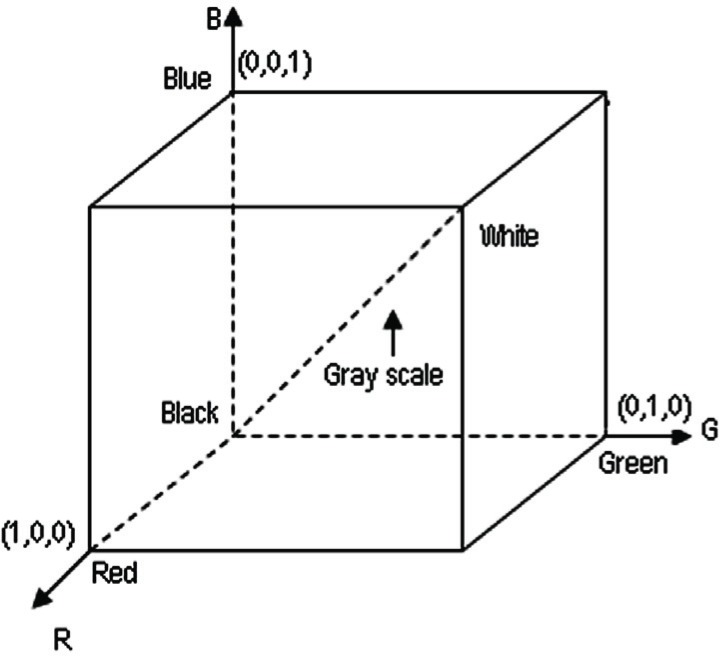
RGB color cube.

A color model is a specification of a coordinate system and a subspace within that system where each color is represented by a single point. In terms of digital image processing, the hardware-oriented models most commonly used in practice are the RGB (red, green, blue) model (9). In RGB model, each color appears in its primary spectral components of red, green and blue. This model is based on a Cartesian coordinate system. The color subspace of interest is the cube shown in Fig. 1 in which RGB values are at three corners; black is at the origin; and white is at the corner farthest from the origin. In this model the gray scale extends from black to white along the line joining these two points. The different colors in this model are point on or inside the cube, and are defined by vectors extending from the origin. For convenience, the assumption is that all color values have been normalized so that the cube is the unit cube.

Images represented in the RGB color model consist of three component images, one for each primary color. The number of bits used to represent each pixel in RGB space is called the pixel depth. Consider an RGB image in which each of the red, green and blue images is an 8-bit image. Under these conditions each RGB color pixel is said to have a depth of 24 bits. The term full-color image is used often to denote a 24-bit RGB color image (10).

The human eye is strongly perceptive to red, green and blue primaries. When humans view a color object, we describe it by its hue, saturation, and brightness. The HSI (hue, saturation, intensity) color model decouples the intensity component from the color-carrying information (hue and saturation) in a color image. Hence the HIS or other similar color models are ideal tools for developing image processing algorithms based on the color descriptions that are natural and intuitive to humans (12).

## METHODS

**Figure 2:**
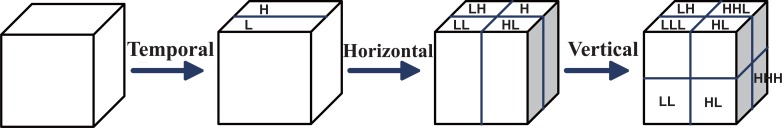
3D decomposition.

3D decomposition consists of performing classic dyadic 2D wavelet decomposition on each image plane followed by 1D dyadic wavelet decomposition in the third direction (6). The 3D decomposition is pictorially depicted in the Fig 2.

For coding color images, each color plane can be treated separately from the other. However, this would not exploit the inter-redundancy between the correlated transformed planes. In fact, two correlated planes with close energy yield similar information of coding in terms of signification when compared to a given threshold. The coder used in this algorithm is 3D-SPIHT which uses a spatial orientation tree structure as illustrated (7).

**Figure 3:**
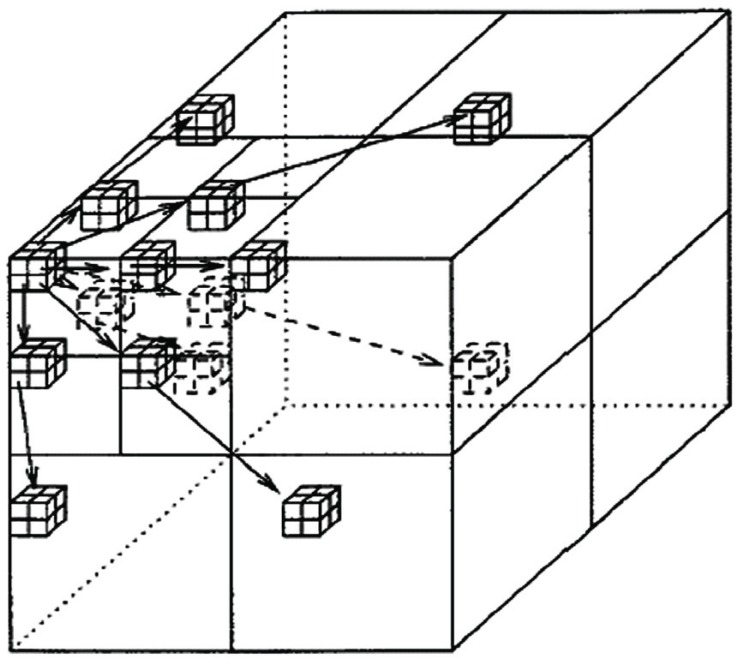
3D Dyadic Tree Structure.

3D SPIHT implementation is very similar to 2-D SPIHT. Sorting of pixels are same as 2-d SPIHT, except with 3-D rather than 2-D trees. Refinement pass is identical, with ordered bit plane transmission of refinement pass. 3D SPIHT results in a completely embedded bit stream. (4) The spatial orientation trees for and 3D are shown in the Fig 3.

Wavelet transforms made a significant contribution in the area of signal and image processing including image coding. The principle behind the wavelet transform is to hierarchically decompose an input signal into a series of successively lower resolution reference signals and their associated detail signals. At each level, the reference signal and detail signal (or signals in the separable multidimensional case) contain the information needed to reconstruct the reference signal at the next higher resolution level (3). Efficient image coding is enabled by allocating bandwidth according to the relative importance of information in the reference and the detail signals and then applying scalar or vector quantization to the transformed data values. The scaling equations on the scaling functions and wavelets show that the decomposition and reconstruction of a signal from a resolution to the next one is implemented by perfect reconstruction filter banks (8).

The reconstruction algorithm provides the coefficients in the finer resolutions. Hence the construction of biorthogonal wavelets is equivalent to the synthesis of perfect reconstruction filters having a stability property. The properties of biorthogonal filters and the effective length of the filters are given in Table 1.

**Table 1:** Properties of Biorthogonal filters

**Family**	Biorthogonal	
**Short name**	Bior	
**Order Nr, Nd**	Nr = 1	Nd = 1,3,5
	Nr = 2	Nd = 2,4,6,8
	Nr = 3	Nd = 1,3,5,7,9
	Nr = 4	Nd = 4
	Nr = 5	Nd = 5
	Nr = 6	Nd = 8
**Support width**	2Nr+1 for rec., 2Nd+1 for dec.	
**Symmetry**	yes	

r, reconstruction; d, decomposition.

### SPIHT Algorithm

**Step 1:** In the array of pixels take log to the base 2 for the pixel with maximum value. Floor the obtained value. Let this Value be n (10).

n= floor (log2 (max {pixel})) (3.41)

**Step 2:** The threshold value is 2^n^.

**Step 3:** Compare the pixel values with the threshold.

- Case A: if the pixel is positive and greater than threshold then it is coded as ‘11’.
- Case B: if the pixel is negative and greater than threshold then it is coded as ‘10’.
- Case C: if the pixel is less than the value of the threshold then it is code as 0.

**Step 4:** The comparison should start from the pixel then to its off-spring and in turn it’s off- spring and so on. After comparing the pixels we put the significant pixels into LSP and insignificant pixels into LIP. The insignificant set is put into LIS.

**Step 5:** Then the value of n is decremented.

**Step 6:** Repeat from step 2.

## IMPLEMENTATION AND RESULTS

The SPIHT algorithm is implemented for the compression of 3-D color volumetric medical images which are in the DICOM standard. 3D images are produced by combining many 2D slices. It is possible to code these 2-D images independently on a slice-by slice basis (11). The 2D slices of volumetric medical images used for the compression algorithm are of size 512 × 512 × 3 each, where the 3 is the color information. The bit depth of the datasets used in this algorithm is 8 bit. Few datasets each consisting 16 of such slices are taken for the implementation of compression technique. The size of each uncompressed dataset is 12 Mb.

The implementation uses the biorthogonal wavelets with 9/7 filter taps for the decomposition of the images.

- Read the 3D color medical image (array of 2D color medical image) in the form of three gray scale images corresponding to red, green and blue components.
- Group the images into three sets of volumetric data corresponding to three color components.
- Apply n level 3D wavelet decomposition (biorthogonal 9/7 wavelet) on each set of volumetric data.
- Apply 3D-SPIHT to three sets of wavelet-decomposed data.

The various test images are shown below from Fig. 4a-Fig. 4d, Fig. 5a-Fig. 5d, Fig. 6a-Fig. 6d and Fig. 7a-Fig. 7d respectively are the dicom image standard and tabulated from Table 2, Table 3, Table 4 and Table 5 correspondingly with various bit rates, PSNR and MSE.

**Fig. 4a:**
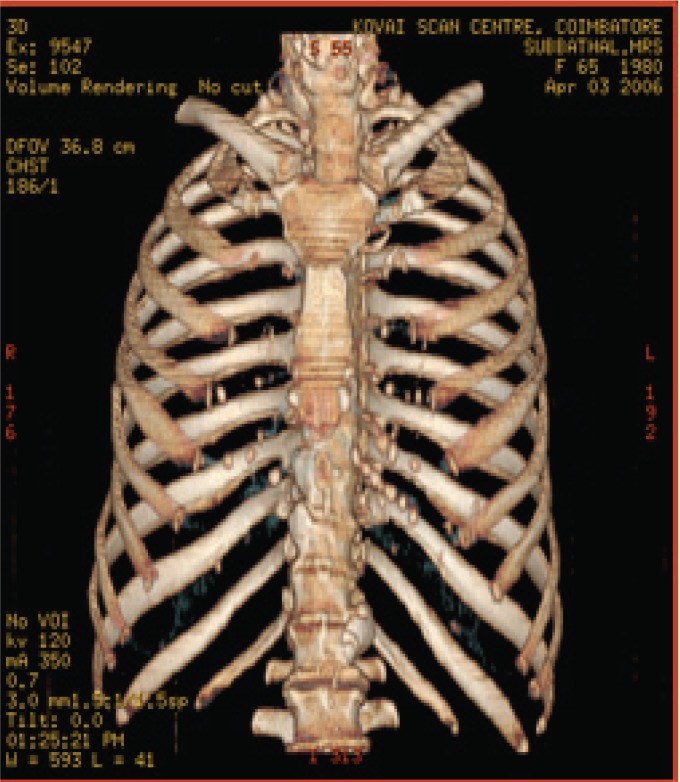
original image

**Fig. 4b:**
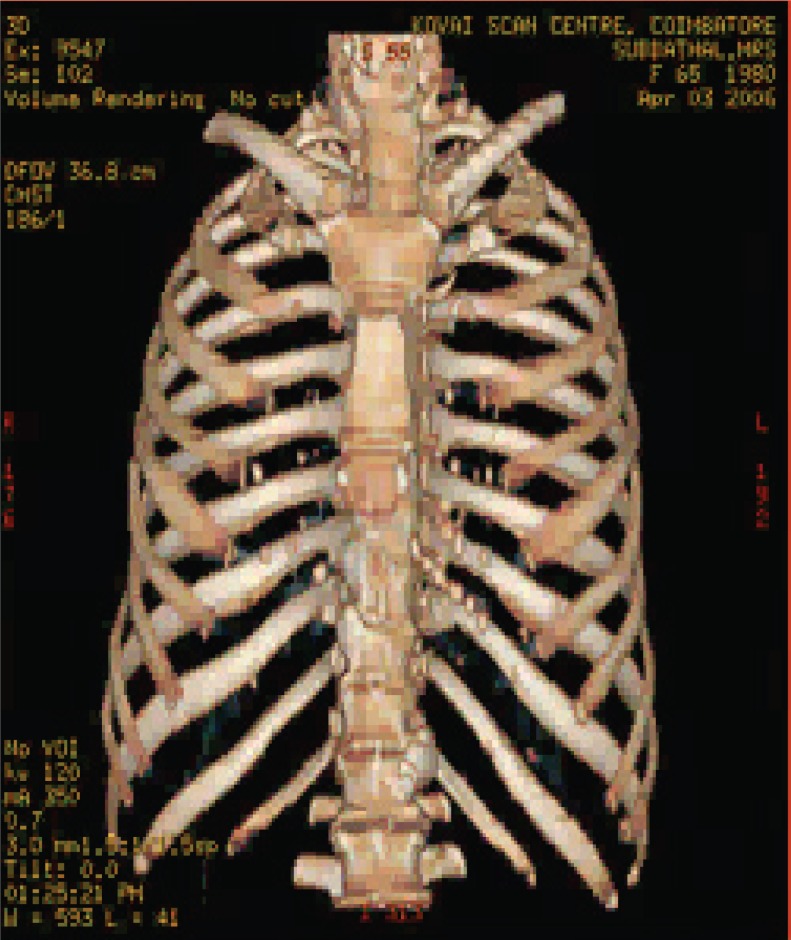
compression at 1.00 bpp

**Fig. 4c:**
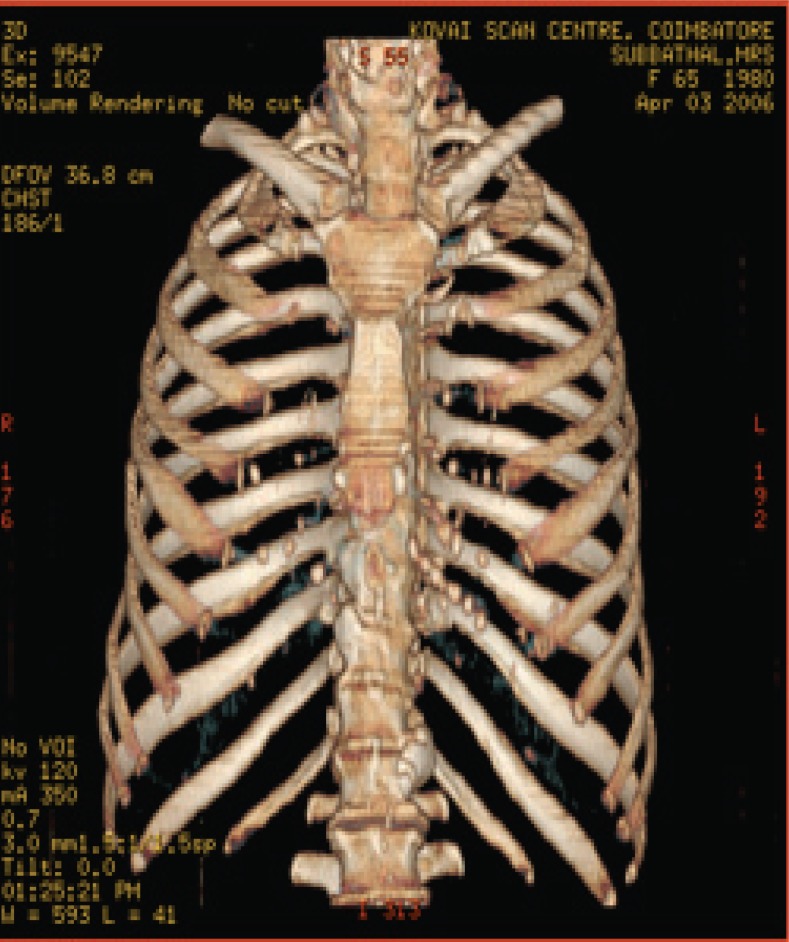
compression at 4.00 bpp

**Fig. 4d:**
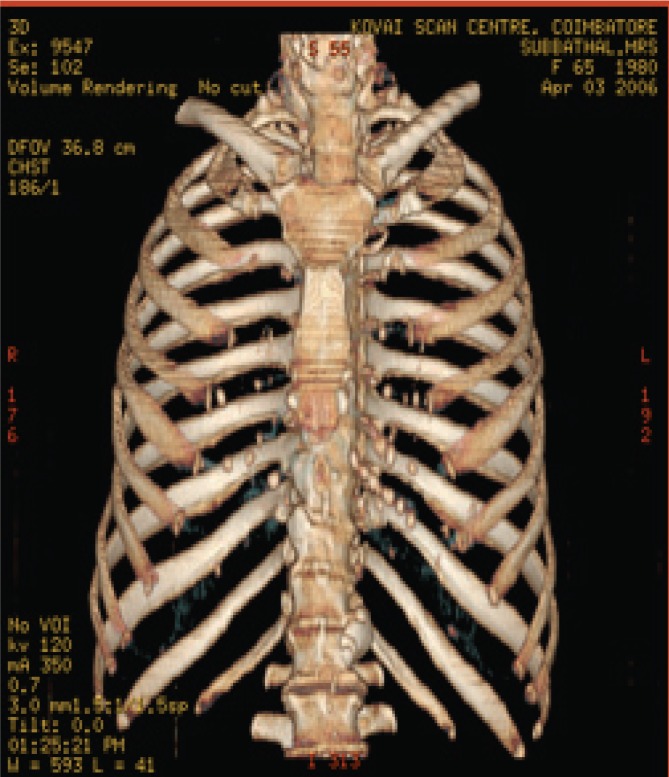
compression at 12.00 bpp

**Table 2:** RESULTS FOR CHEST IMAGE

Figures	Rate in bpp	MSE	Cr	PSNR

Fig. 4b	1	80.7231	93.75	77.2596
Fig. 4c	4	0.2980	75	101.5872
Fig. 4d	12	1.42E-04	25	134.794

**Fig. 5a:**
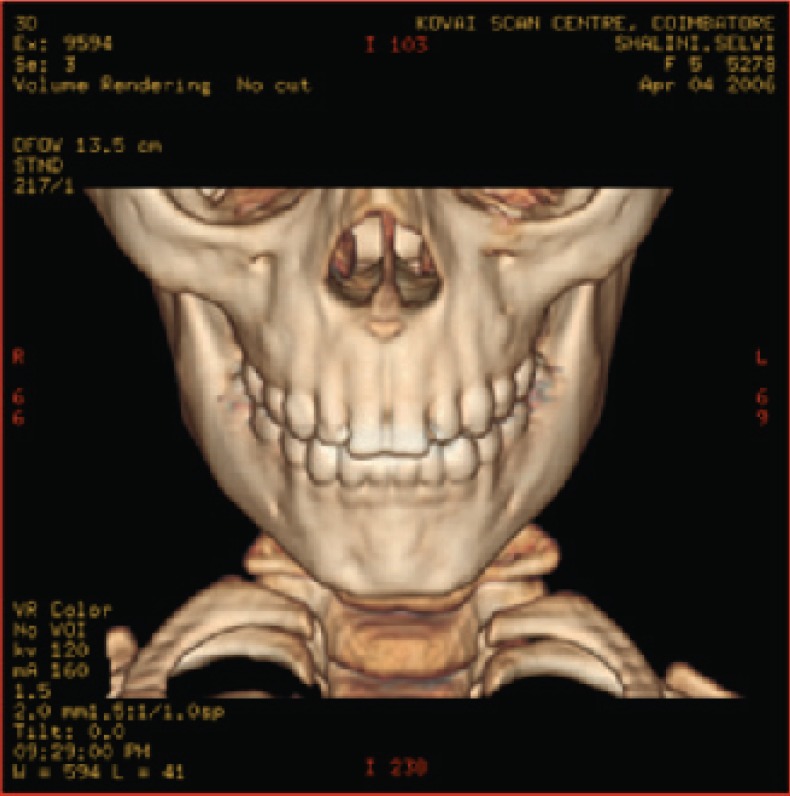
original image

**Fig. 5b:**
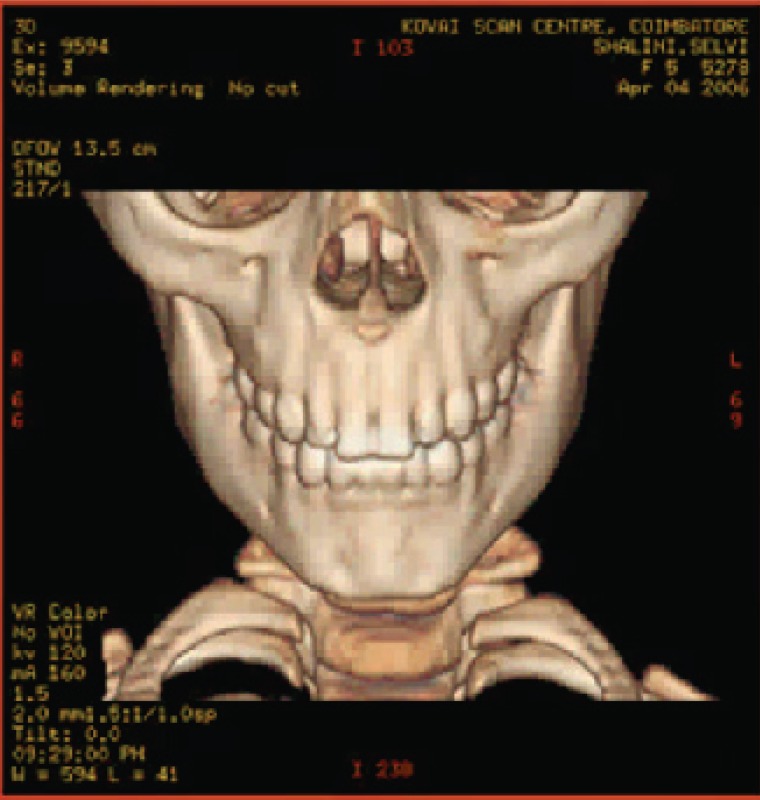
compression at 1.00 bpp

**Fig. 5c:**
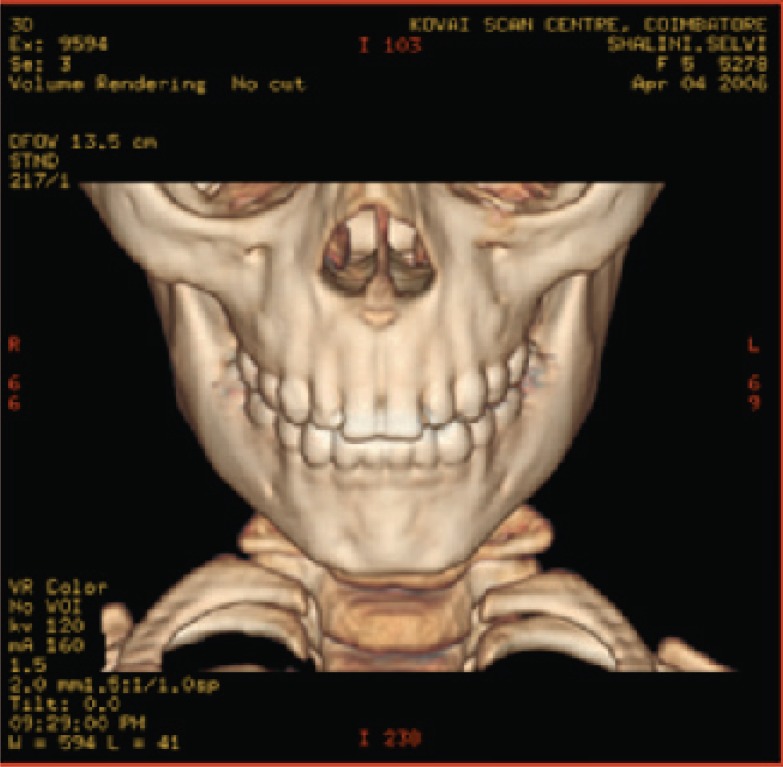
compression at 4.00 bpp

**Fig. 5d:**
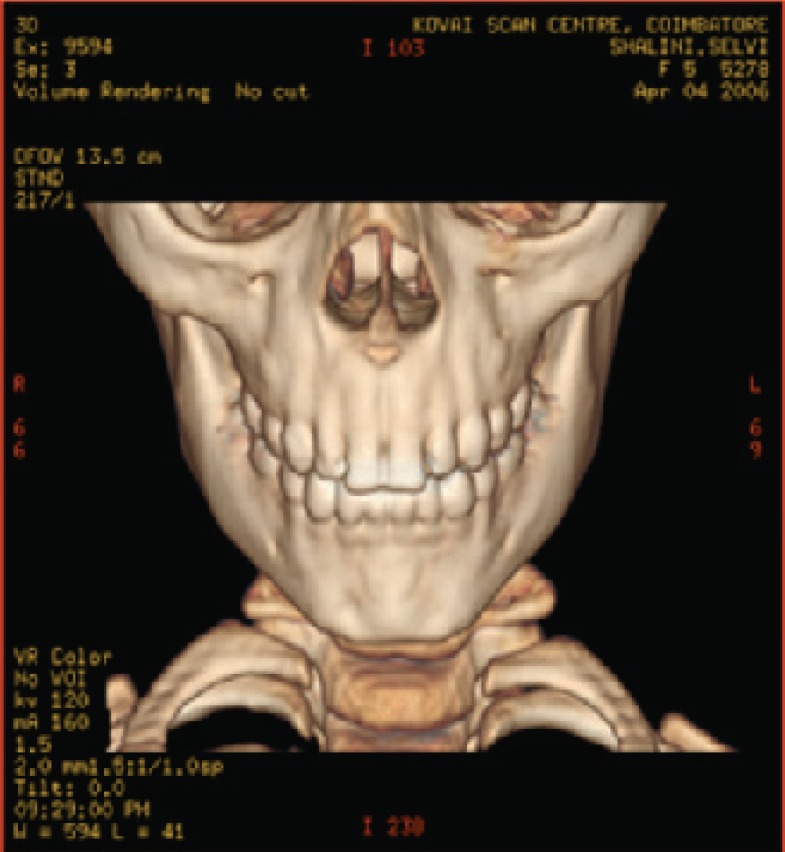
compression at 12.00 bpp

**Table 3:** RESULTS FOR SKULL IMAGE

Figures	Rate in bpp	MSE	Cr	PSNR

Fig. 5b	1	20.166	93.75	83.283
Fig. 5c	4	0.005	75	119.312
Fig. 5d	12	6.063E-09	25	178.503

**Fig. 6a:**
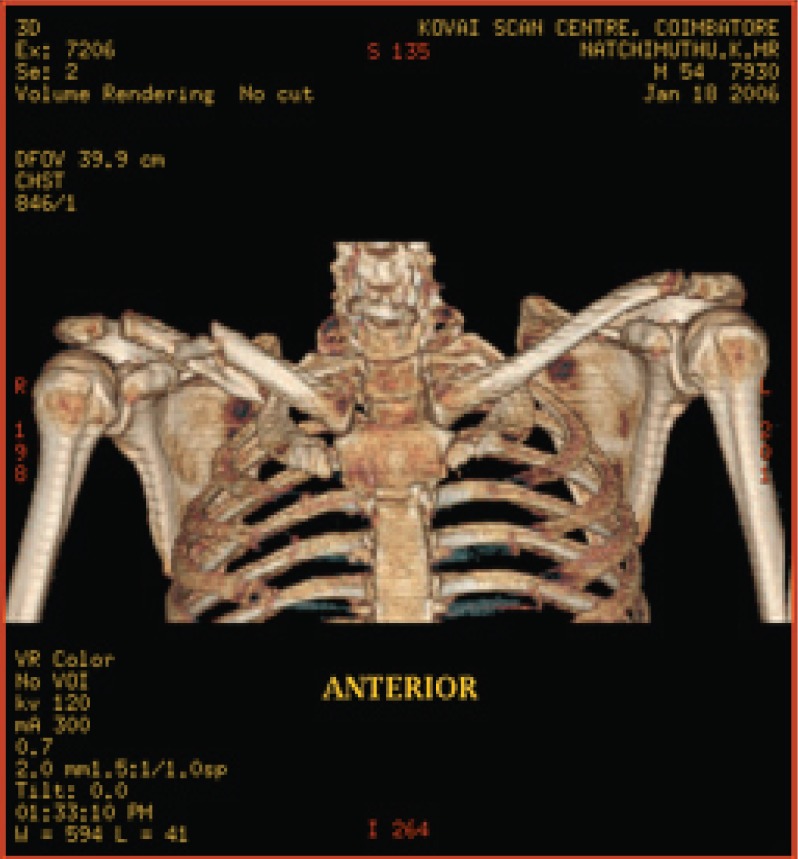
original image

**Fig. 6b:**
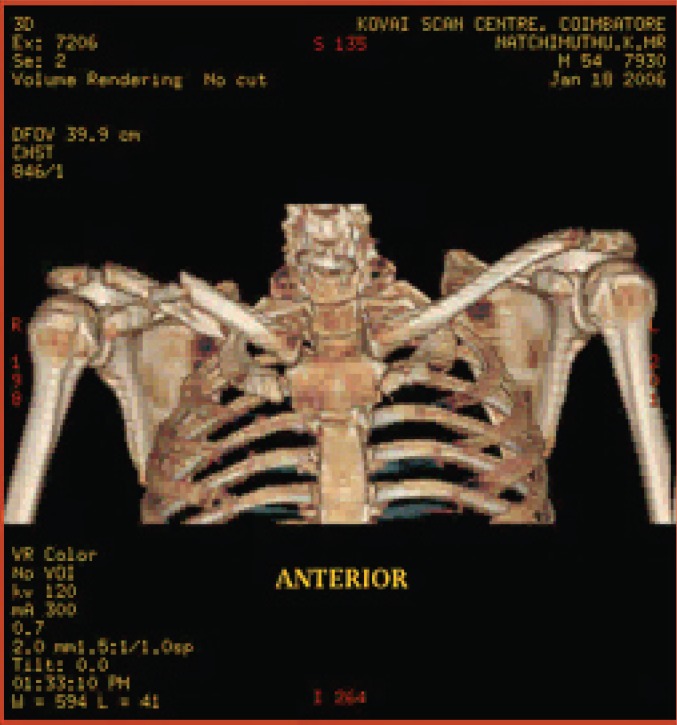
compression at 1.00 bpp

**Fig. 6c:**
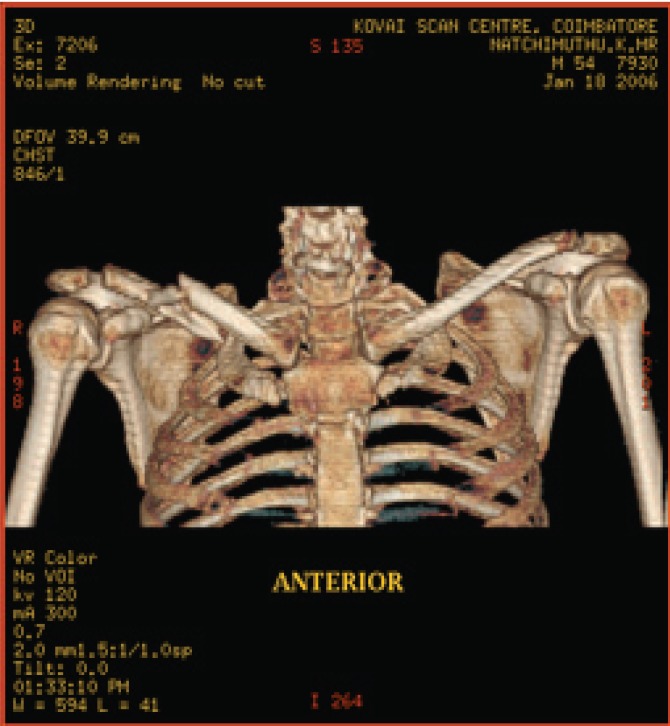
compression at 4.00 bpp

**Fig. 6d:**
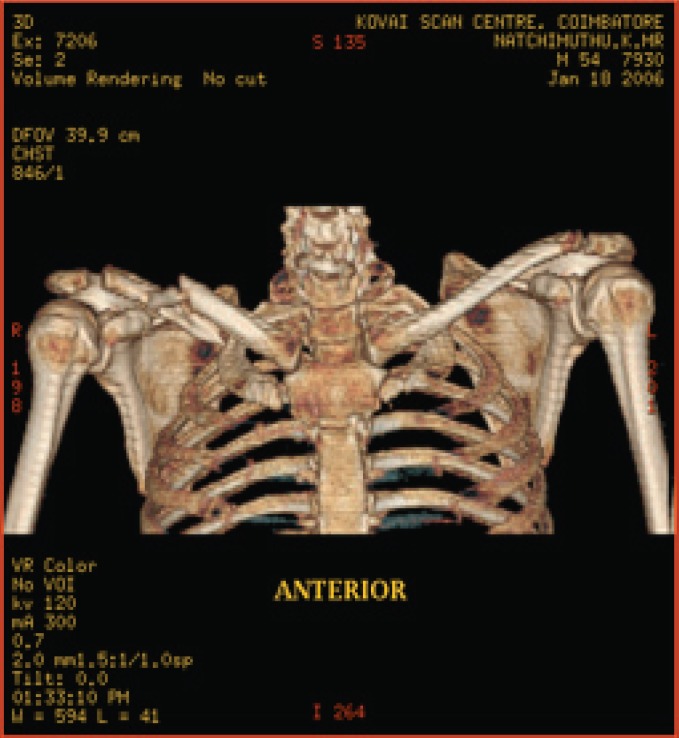
compression at 12.00 bpp

**Table 4:** RESULTS FOR SHOULDER IMAGE

Figures	Rate in bpp	MSE	CR	PSNR

Fig. 6b	1	28.5680	93.75	81.7708
Fig. 6c	4	0.0511	75	109.2454
Fig. 6d	12	2.03E-05	25	143.245

**Fig. 7a:**
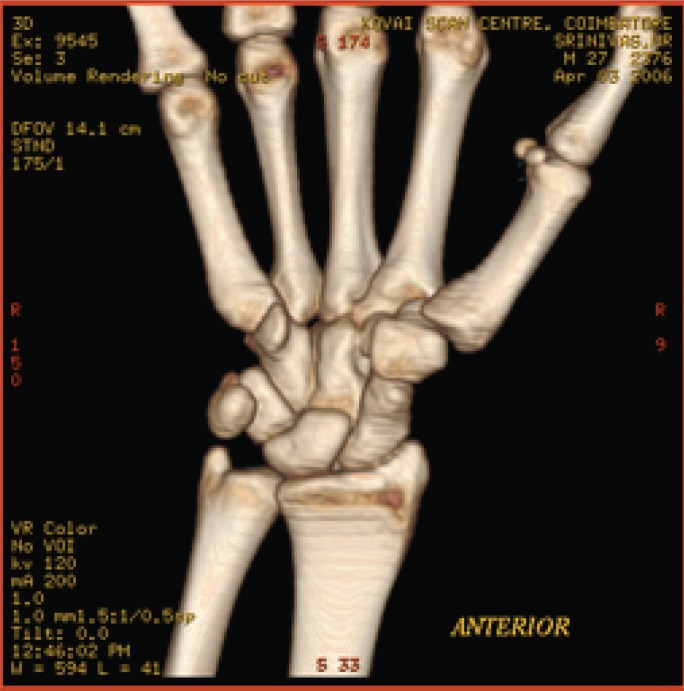
original image

**Fig. 7b:**
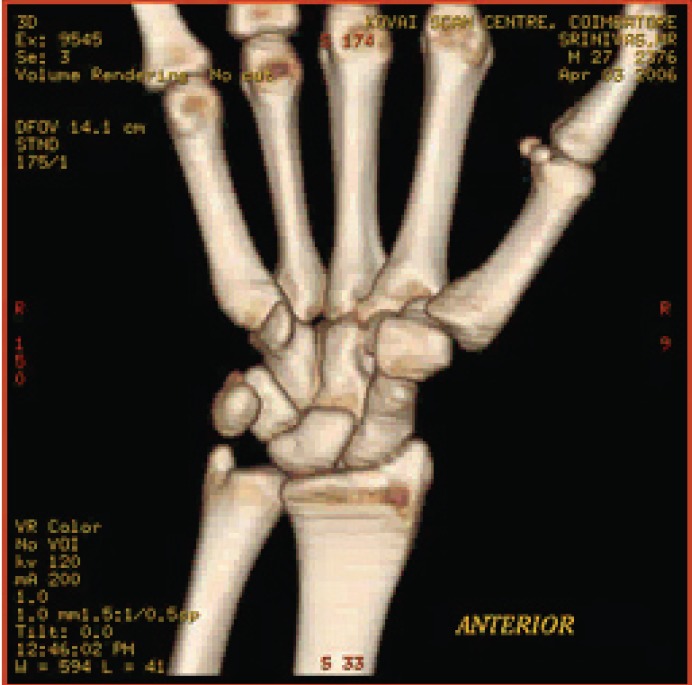
compression at 1.00 bpp

**Fig. 7c:**
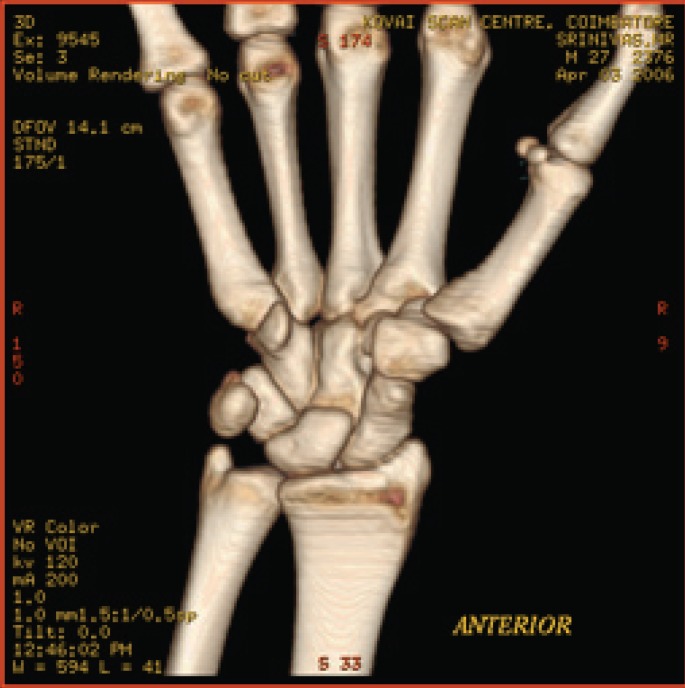
compression at 4.00 bpp

**Fig. 7d:**
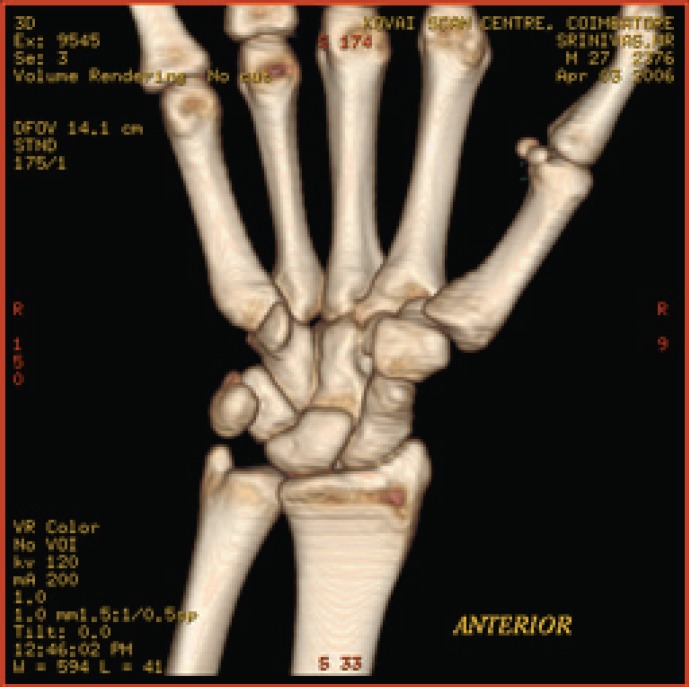
compression at 12.00 bpp

**Table 5:** RESULTS FOR WRIST IMAGE

Figures	Rate in bpp	MSE	CR	PSNR

Fig. 7b	1	19.6206	93.75	83.4025
Fig. 7c	4	0.05	75	109.340
Fig. 7d	12	7.32E-09	37.5	177.684

Three Dimensional SPIHT is implemented for (512 × 512 × 64) voxels 16-bit 3D color images, (256 × 256 × 32) voxels 16-bit 3D color images, (512 × 512 × 64) voxels 16-bit 3D gray images, (256 × 256 × 32) voxels 16-bit 3D gray images. The parameters such as MSE, CR and PSNR for different bit rates have been tabulated. The parameters such as MSE, CR and PSNR for different bit rates have been tabulated. The Table 6 reveals that the compression ratio and PSNR are better than JPEG 2000 compression (2).

**Table 6:** Comparison of 3D DCT, 3D JPEG2000 and 3D SPIHT

3D DCT			3D JPEG 2000 (2)			3D SPIHT		
Uncompressed image size in MB	Compressed image size in MB	CR in %	Uncompressed image size in MB	Compressed image size in MB	CR in %	Uncompressed image size in MB	Compressed image size in MB	CR in %

134.2	93.9	30	224	75.8	66	134.2	25.5	81
268.4	142.2	47	310	120	61	268.4	67.1	75

This tabulation reveals that the compression ratio and PSNR are better than JPEG 2000 compression (2).

## PACS

In medical imaging, picture archiving and communication systems (PACS) are computers or networks dedicated to the storage, retrieval, distribution and presentation of images. The medical images are stored in an independent format. The most common format for image storage is DICOM (Digital Imaging and Communications in Medicine). PACS replaces hard-copy based means of managing medical images, such as film archives (13). It expands on the possibilities of such conventional systems by providing capabilities of off-site viewing and reporting (distance education, tele-diagnosis). Additionally, it enables practitioners at various physical locations to access the same information simultaneously, (teleradiology). With the decreasing price of digital storage, PACS systems provide a growing cost and space advantage over film archives. PACS is offered by virtually all the major medical imaging equipment manufacturers, medical IT companies and many independent software companies. The goals are to improve patient care, maximize limited resources, and realize cost savings.

## CONCLUSION

The results obtained using the proposed compressed algorithm show that the performance of the biorthogonal filters with filter taps 9/7 is better than those obtained using filters with filter taps 5/3 and 9/11 for the compression of the volumetric color medical images. The proposed algorithm gives better results than that of the existing JPEG 2000. From this we conclude that the proposed 3D-SPIHT algorithm gives better performance than JPEG 2000 in terms of Compression Ratio and PSNR.
